# Therapeutic efficacy of *n*-Docosanol against velogenic Newcastle disease virus infection in domestic chickens

**DOI:** 10.3389/fmicb.2022.1049037

**Published:** 2022-11-22

**Authors:** Ahmed Orabi, Ashraf Hussein, Ayman A. Saleh, Ayman M. Megahed, Mohamed Metwally, Hassan Moeini, Aya Sh. Metwally

**Affiliations:** ^1^Department of Virology, Faculty of Veterinary Medicine, Zagazig University, Zagazig, Egypt; ^2^Institute of Virology, School of Medicine, Techenical University of Munich, Munich, Germany; ^3^Department of Avian and Rabbit Medicine, Faculty of Veterinary Medicine, Zagazig University, Zagazig, Egypt; ^4^Department of Animal Wealth Development, Genetics and Genetic Engineering, Faculty of Veterinary Medicine, Zagazig University, Zagazig, Egypt; ^5^Department of Veterinary Public Health, Faculty of Veterinary Medicine, Zagazig University, Zagazig, Egypt; ^6^Department of Pathology, Faculty of Veterinary Medicine, Zagazig University, Zagazig, Egypt; ^7^Department of Pharmacology, Factulty of Veterinary Medicine, Aswan University, Aswan, Egypt

**Keywords:** ND, *n*-Docosanol, therapy, virus load, virus shedding, domestic chickens

## Abstract

**Introduction:**

The control of Newcastle disease virus (NDV) infection depends solely on vaccination which in most cases is not sufficient to restrain the consequences of such a highly evolving viral disease. Finding out substances for preparing an efficient anti-ND drug would be of high value. *n*-Docosanol is a saturated fatty alcohol with an inhibitory effect against many enveloped viruses. In this study, we evaluated the therapeutic effect of *n*-docosanol on NDV infection and shedding in chickens.

**Methods:**

Chickens infected with a highly virulent NDV were treated with low to high concentrations of *n*-docosanol (20, 40, and 60 mg/kg body weight) for 4-successive days, once they showed the disease symptoms. Survival and curative rates, virus load, histopathological scoring, and virus shedding were defined.

**Results:**

Symptoms development was found to discontinue 24–72 hours post-treatment. Survival rate in the NDV-infected chickens raised 37.4–53.2% after the treatment. *n*-Docosanol treatment was also found to significantly reduce virus load in the digestive (26.2–33.9%), respiratory (38.3–63%), nervous (26.7–51.1%), and lymphatic (16.4–29.1%) tissues. Histopathological scoring of NDV lesions revealed prominent rescue effects on the histology of different tissues. Importantly, *n*-docosanol treatment significantly reduced virus shedding in oropharyngeal discharge and feces thereby allowing the restriction of NDV spread.

**Conclusion:**

Our findings suggest *n*-docosanol as a promising remedy in the control strategy of Newcastle disease in the poultry industry.

## Introduction

Newcastle disease (ND) is a highly contagious and devastating disease of birds. It is considered one of the major infectious obstacles competing for the development of the poultry industry globally, especially in endemic countries. *Despite* the *availability* of *vaccines* against NDV, disease outbreaks continue to occur frequently, even in vaccinated birds. Consequently, ND is still listed among the *most* damaging disease in the poultry industry (Zhang et al., 2011; OIE, 2012). There is no treatment for this disease, and NDV vaccination is mostly ineffective against highly virulent strains. Furthermore, post-vaccinal consequences, especially in the case of immunosuppression and during winter, is of high significance (Chang and Dutch, 2012). This asserts the urgent need for an alternative approach like new antivirals to control such a serious disease.

*n*-Docosanol is a long-chain (C-22) saturated fatty alcohol with antiviral activity against herpes simplex virus (HSV) which has been approved by the Food and Drug Administration (FDA) as a pharmaceutical drug against cold sores caused by HSV (Abreva, 2018). *n*-Docosanol has also shown a potent inhibitory effect against a wide range of enveloped viruses including cytomegalovirus, human immunodeficiency virus (HIV), respiratory syncytial virus (RSV), influenza A virus, and murine Friend leukemia virus (Katz et al., 1994; Marcelletti et al., 1996). *n*-Docosanol is characterized by the lack of toxicity, mutagenicity, or teratogenicity on eukaryotic cells (Snipes et al., 1977; Johnson, 1988), even at high concentrations (up to 300 mM), making it a favorable therapeutic alternative for viral diseases (Katz et al., 1991). Interestingly, *n*-docosanol has been shown to be endogenously produced in the human body (Nicolaides, 1967; Harvey et al., 1987; Harvey, 1989); *n*-docosanol has been also found in many natural foodstuffs such as olive oil, fish oil, sunflower seeds, lettuce leaves, beeswax, and apples (Radler, 1965; Tulloch, 1976; Morrison, 1983; Frega et al., 1993). Although some studies showed that *n*-docosanol has no direct antiviral effect (Katz et al., 1991), it has been to undergo a metabolic conversion intracellularly preventing virus entry and consequently allowing the cells to abort the infectious cycle of many enveloped viruses (Katz et al., 1994; Pope et al., 1996). *n*-Docosanol can inhibit infections caused by enveloped viruses that induce both types of cell fusion, fusion from without (FFWO) or fusion from within (FFWI) (Katz et al., 1994; Marcelletti et al., 1996). In the present study, we assessed the therapeutic efficacy of *n*-docosanol against NDV infection and shedding in chickens.

## Materials and methods

### Virus, antiviral drug, animal

The virulent genotype VIId NDV strain (NDV/chicken/Egypt/Sharkia14/2016) under the accession number KY075886 (Orabi et al., 2017) was used in this study. Virus titration was carried out according to Reed and Muench (Reed and Muench, 1938). *n*-Docosanol (Sigma Aldrich, Germany) was used as an antiviral drug against NDV infection in chickens. The drug was dissolved in 1% preheated (50°C) Poloxamer 188 (Sigma Aldrich, United Kingdom) by sonication at an initial output of 65 W for 21 min to get a final concentration of 100 mg/ml according to Katz et al. (1991). Specific-pathogen-free (SPF) chickens from Cobb-500 parents were used for the *in-vivo* experiment. The chickens were reared on disinfected fresh saw dust deep litter in the isolation rooms (Faculty of Veterinary Medicine, Zagazig University, Egypt). Feed and water were manually provided in feeders and waterers *ad libitum* during the rearing period. A final stocking density of 9 birds/m^2^ was achieved by the 20th day of age. All the procedures were conducted according to the international guiding principles for biomedical research involving animals and after being reviewed and approved by the Zagazig University − Institutional Animal Care and Use Committee (ZU-IACUC) under the approval number ZU-IACUC/2/F/128/2020.

After a pilot study using 10 different dosages of *n*-docosanol (10–100 mg/kg.body weight (BW)), three different dosages 20, 40, and 60 mg/kg BW were selected as low, medium and high dosages for the study. Once the clinical signs of ND started to appear (51 h post-infection, on the 23^rd^ day old), each dosage was injected intramuscularly in the thigh muscle and repeated for 3 successive days, on days 24, 25, and 26.

### *In vivo* experiment

**Figure 1:**
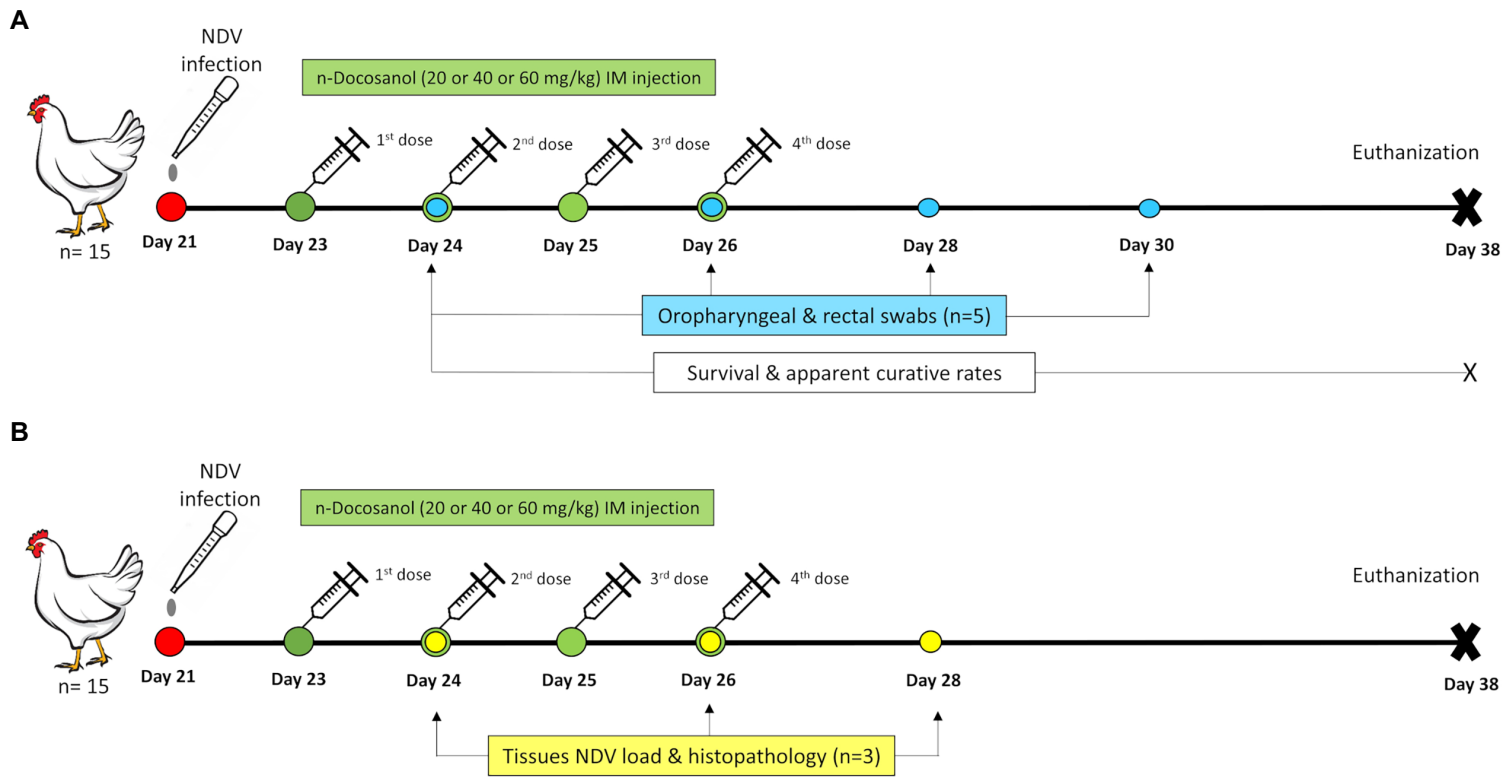
Schematic illustration of the experimental timeline for the chickens treated with *n*-docosanol. **(A)** The timeline of chicken groups used for the assessment of survival and curative rates and NDV shedding post treatment. **(B)** The timeline of chicken groups used for the assessment of tissues’NDV load and histopthology post treatment.

One-day-old SPF chicks (*n* = 140) were divided randomly into two major groups, one for recording clinical signs, postmortem lesions, survival rate, and apparent curative rate and the other for recording tissues’ virus load and histopathological scoring (Figure 1). Both major groups were sub-divided randomly into four subgroups: all the chicks in these subgroups were first infected *via* the occulo-nasal route on day 21 with a viral dose of 10^6.3^ EID_50_ (100 μl) (Ferreira et al., 2019). Chickens in subgroups 1, 2, and 3 were then treated with high (60 mg/kg), medium (40 mg/kg), and low (20 mg/kg) dosages of *n*-docosanol, respectively. Chicks in subgroup 4 were not received the drug. The last 20 chickens, which served as a negative control group, were administered intramuscularly with 1% Poloxamer 188 alone.

### Virus infection evaluation post *n*-docosanol treatment

#### Clinical and postmortem examination

The chickens were observed and examined intermittently every day post-infection until the end of *in vivo* experiment (38 days). The obtained results for (i) mortality, (ii) body weight, (iii) digestive signs (diarrhea) and postmortem lesions (enteritis, intestinal ulcers, and hemorrhages on proventriculus and cecal tonsils), (iv) respiratory signs (gasping, sneezing, coughing, and rales) and postmortem lesions (congestion of lungs and respiratory tracts), and (v) neurological signs (nervous manifestation including tremors, paralyzed wings and legs, circling, and torticollis) and *post mortem* lesions (brain congestion and hemaorrhages) were recorded post the first *n*-docosanol dosage injection on day 24.

#### Assessment of survival rate

The survival rate of chickens in each group was calculated and analyzed by the survival curve estimator of GraphPad Prism version 6.01. The number of live chickens in each group was recorded at 1–15 days post the first *n*-docosanol dosage injection.

#### Assessment of the apparent curative rate

The apparent curative rate of chickens in each group was calculated by the following formula:

Apparent curative rate = the number of chickens without ND clinical signs / the total number of chickens per each group.

The number of chickens without ND clinical signs in each group was recorded at 1–15 days post the first *n*-docosanol dosage injection.

#### Assessment of tissues’ NDV load

Tissues’ NDV titer was assessed in the lymphatic (spleen), digestive (proventriculus), respiratory (lungs), and nervous (brain) tissues. Three chickens from each group were randomly selected and humanely sacrificed on days 1, 3, and 5 post the first treatment with *n*-docosanol. The organs were harvested and then homogenized in a sterilized phosphate-buffered saline (PBS, pH 7.4) supplemented with 50 U/ml Penicillin–Streptomycin mixture (PenStrep, Lonza) in a ratio of 1:5 (w/v). Following three sequential steps of freezing and thawing, the homogenates were centrifuged at 5000 × *g* for 30 min at 4°C, and the clear supernatants were subjected to virus titration by real-time qRT-PCR.

#### Histopathological scoring

The next day after the last *n*-docosanol dosage injection, five chickens from each group (freshly dead or alive) were randomly selected and sacrificed for standardized necropsy procedures according to Lucio-Martínez and Korich (Lucio-Martínez and Korich, 2010). To this end, representative tissue specimens from the brain, liver, lungs, spleen, proventriculus, and cecal tonsils were harvested and immediately fixed in 10% neutral buffered formalin solution for 48 h. The specimens were then trimmed, washed, dehydrated in graded ethanol, cleared in xylene, impregnated, and embedded in paraffin wax. Thereafter, at 4–5 μm thickness tissue sections were stained with hematoxylin and eosin following the protocol described by Suvarna et al. (2018), and then were examined under the microscope. Subsequently, multiparametric multiorgan quantitative lesion scoring was carried out for all the groups according to Hussein et al. (Hussein et al., 2018). Briefly, 10 randomly selected nonoverlapped snapshots with a fixed size (50 images/organ/group) were taken using the AmScope digital imaging system. The images were then analyzed for lesions’ severity and frequency. Lesions’ severity was determined using a five-point scale as follows: zero, absence of the lesion; 1, focal distribution; 2, multifocal distributions; 3, locally extensive distribution; 4, diffuse distribution. Lesions’ frequency was determined using the following formula: FQ (%) = Nlesion × Ntotal-1 × 100. Where, Nlesion and Ntotal represent the total number of images exhibited a lesion, and the total number of images in the group (50), respectively.

### NDV shedding evaluation post *n*-docosanol treatment

Virus shedding was determined in oropharyngeal (*n* = 5) and cloacal (*n* = 5) swab samples on days 1, 3, 5, and 7 days post the first *n*-docosanol dosage treatment by real-time qRT-PCR. Infectivity of the recovered viruses from swab samples was also assessed in SPF embryonated chicken eggs (SPF-ECEs) by calculating the EID_50_ after a standardization step using serial dilutions (10-fold) of the RNA extracted from the applied NDV strain. The standard curve was used to define the detection limit of the individual qRT-PCR run. The results were expressed as EID50/ml equivalents.

### NDV titration by real-time qRT-PCR

The total viral RNA was extracted from the samples using QIAamp Viral RNA extraction Kit (Qiagen, United States) following the manufacturer’s instructions. Specific primers (forward; 5’-AGTGATGTGCTCGGACCTTC-3′ & reverse; 5’-CCTGAGGAGAGGCATTTGCTA-3′) and probe (5′-[FAM]TTCTCTAGCAGTGGGACAGCCTGC[TAMRA]-3′) amplifying and detecting a highly conserved sequence within NDV M gene viral RNAs (Wise et al., 2004) using the Qiagen one-step RT-PCR Kit. The RT-PCR thermal profile included an initial RT step at 50°C for 30 min followed by 15 min treatment at 95°C. The PCR cycling profile consisted of 40 cycles of (i) denaturation at 94°C for 10 s, (ii) annealing at 52°C for 30 s, and (iv) extension at 72°C for 10 s, with a final extension step at 72°C for 10 min. The quantification of the extracted RNAs was carried out on the Rotor Gene Q 2plex Real Time Pcr System (Qiagen, United States).

### Statistical analysis

Kaplan–Meier estimator with Logrank test of GraphPad Prism version 6.01 was used to compare the numbers of survived chickens post-challenge. One-way ANOVA with Tukey’s multiple range test was used to compare the percent of apparently healthy chickens post-challenge and treatment and also to compare between the mean of log_10_ qRT-PCR titers of both tissues and swabs recovered virus.

## Results

### *n*-docosanol treatment reduced NDV clinical signs and postmortem lesions in chickens

The ND clinical signs and postmortem lesions were recorded 24 h post the first *n*-docosanol dosage injection and until the end of the experiment. The negative control group appeared normal throughout the experiment, while in NDV-infected groups, few chickens (up to 2 chickens within each group) started to develop slight ND symptoms (slight greenish diarrhea, conjunctivitis, and/or slight gasping) 51 h post infection. One day post the first *n*-docosanol dosage injection, infected chickens showed collectively much less clinical signs and postmortem lesions as compared to the untreated infected group which showed moderate to severe clinical signs and post-mortem lesions with 100% mortality within 7 days post-infection. Unlike the untreated group, a significant reduction in mortalities (46.7–66.7%) was obtained in the treated groups (Table 1). Relatively low loss in body weight (12.3–22%) was observed in the treated chickens when compared to the negative control group (Table 1). No significant differences in mortality, bodyweight loss, and clinical signs and lesions were detected between the chickens treated with the high and medium dosages of *n*-docosanol. In contrast, the chickens treated with the low dosage of *n*-docosanol showed a relatively higher percentage of mortality, body weight loss, and clinical signs (Table 1). The most predominant clinical signs and postmortem lesions in the treated groups were digestive (58.35% for both groups) then the respiratory (48.33 and 40.02%, respectively), and finally the neurological (16.65 and 20%, respectively; Table 1).

**Table 1:** Effect of 20, 40, and 60 mg/k.g BW *n*-docosanol treatment on the mortality percent, final body weight gain, clinical signs, and postmortem lesions of experimentally NDV-infected chickens.

**Dose** (mg/kg)	**NDV infection**	**Mortality** (%)	**Body weight** (gm)	**Digestive**		**Respiratory**		**Neurological**	
				**Signs** (%)	**Lesions** (%)	**Signs** (%)	**Lesions** (%)	**Signs** (%)	**Lesions** (%)
60	+	46.7	1857	46.7	40	40	33.3	6.7	6.7
40	+	53.3	1861	46.7	46.7	40	33.3	13.3	13.3
20	+	66.7	1,656	60	60	53.3	46.7	13.3	13.3
−	+	100	NS	80	66.7	60	46.7	33.3	46.7
−	−	0	2,122	0	0	0	0	0	0

NS, no survivors.

### *n-*docosanol treatment increased survival and apparent curative rates

**Figure 2:**
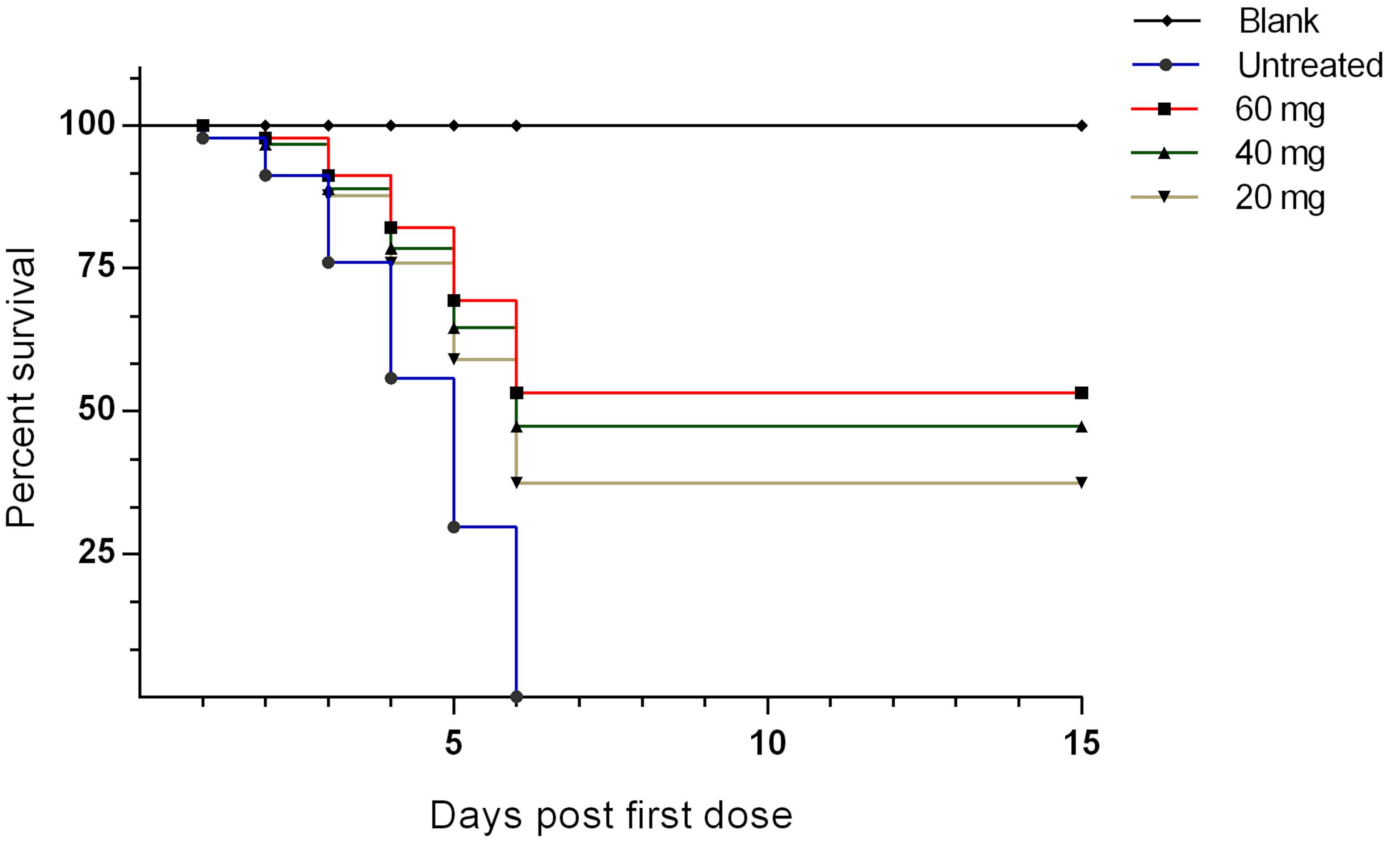
The survival rate of experimentally NDV infected chickens during and after treatment with 20, 40, and 60 mg/k.g BW *n*-docosanol. The number of alive chickens in each group was recorded at 1–15 days post the first *n*-docosanol dosage injection. The survival rate was graphed using GraphPad Prism version 6.01 and analyzed by Kaplan–Meier estimator with Logrank test. A significant difference (*value of p* of <0.001) appeared between the untreated group and both the high (60 mg/kg) and medium (40 mg/kg) dosage groups.

The survival rate was calculated in each group for 15 days post the first *n*-docosanol injection. The data showed 1 day delay in starting death in the *n*-docosanol-treated groups, as compared to the untreated. On day 6 post-first *n*-docosanol treatment no more death was observed in the treated groups, however, the death stopped relatively later (10–11 h) on day 6 in the low-dosage treated group (data not shown). In contrast, no chicken survived in the untreated group (Figure 2). The treated groups showed a survival rate of 33.33–53.33%. The highest survival rate was detected in the groups treated with the high dosage of *n*-docosanol, decreasing from 93.33% on day 2 post-treatment to 53.33% on day 6 post-treatement, while the survival rate of the untreated group fell from 86.67% on first-day post-infection to 0% on day 6. The medium-dosage treated group showed a survival rate close to that of the high-dosage treated one (decreasing from 93.33% at day 2 to 46.67% at day 6 post-first injection), while the low-dosage treated group exhibited a lower survival rate, decreasing from 86.67% on day 2 to 33.33% on day 6 post first injection (Figure 1).

**Figure 3:**
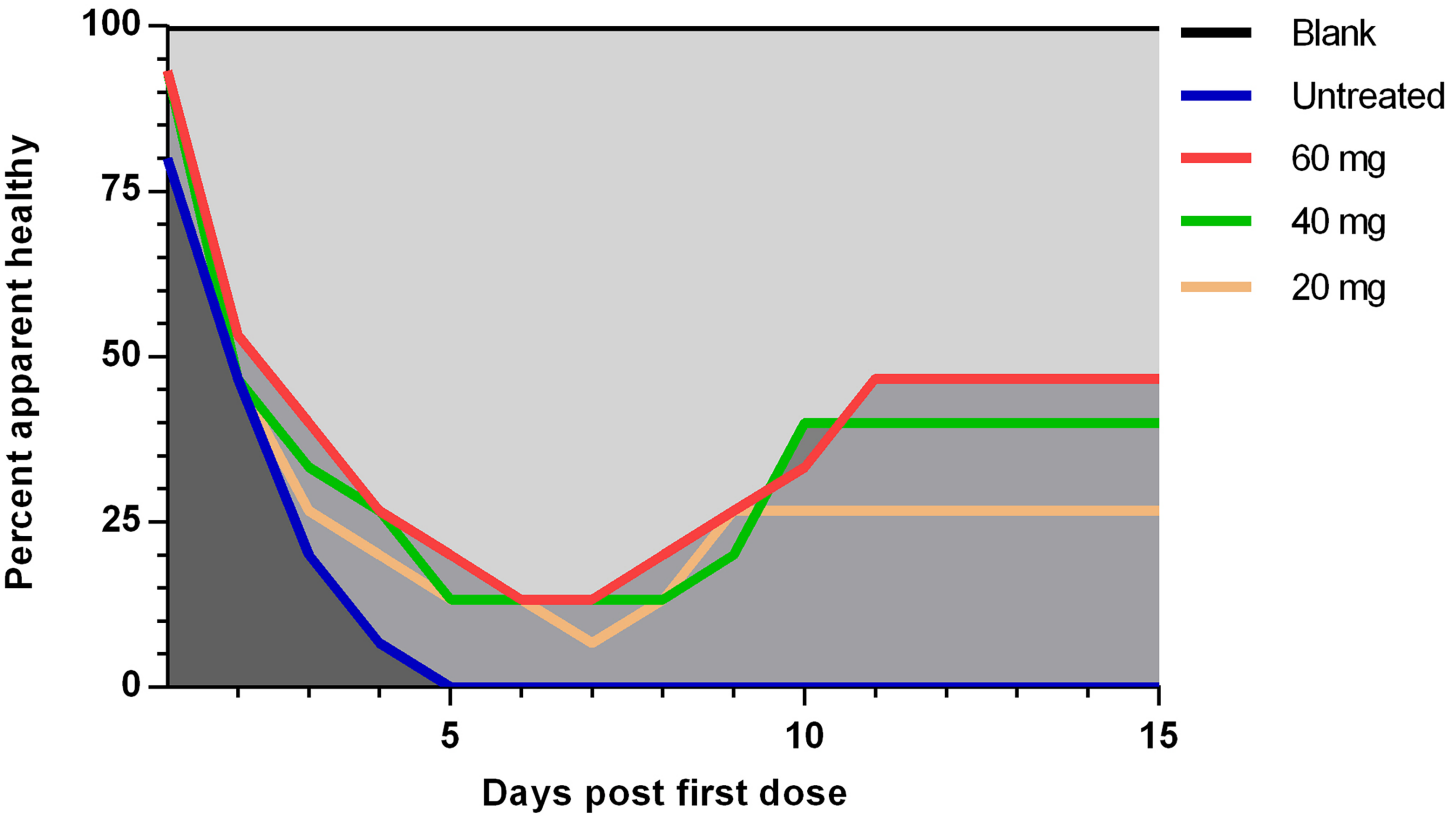
The apparent curative rate of experimentally NDV infected chickens during and after treatment with 20, 40, and 60 mg/k.g BW *n*-docosanol. The number of chickens without ND clinical signs in each group was recorded at 1–15 days post the first *n*-docosanol dosage injection. The apparent curative rate was calculated by the formula: Apparent curative rate = the number of chickens without ND clinical signs / the total number of chickens per group. One-way ANOVA with Tukey’s multiple range test was used to compare between the groups. A significant difference (*value of p* of <0.05) appeared between the untreated group and both the high (60 mg/kg) and medium (40 mg/kg) dosage groups.

The curative rate was also calculated for each group at days 1–15 post the first dosage injection. One-fifth (20%) of the chickens in the untreated group developed ND signs at day one post-treatment which increased rapidly on the successive days until all the chickens (100%) showed the signs at 5 days (Figure 3). Then after, all the chickens died on the next day (Figure 2). In contrast to the low-dosage treated group both high- and medium-dosage treated groups displayed a significant (*p* < 0.05) increase in the apparent curative rates. The percent of apparently healthy chickens in the both groups decreased gradually from the first day after the first dosage injection and reached its lowest degree (13.33%) on days 7 and 8, and then reached its highest level (33.33–40%) on days 9–11 (Figure 3).

### *n*-docosanol treatment reduced virus load in tissues

**Figure 4:**
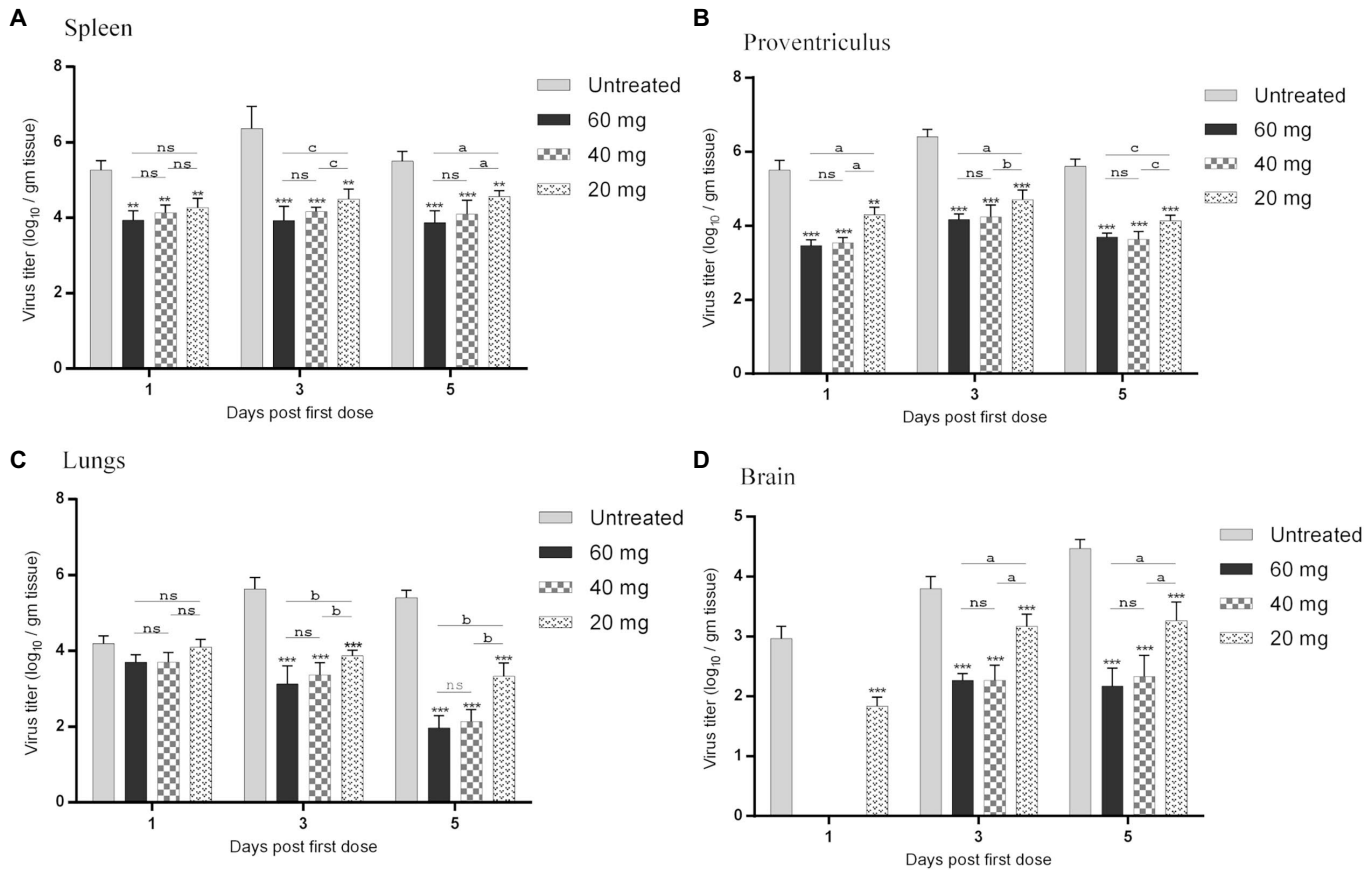
NDV load in organs resembling the **(A)** lymphatic (spleen), **(B)** digestive (proventriculus), **(C)** respiratory (lungs), and **(D)** nervous (brain) systems of experimentally infected chickens during and after treatment with 20, 40 and 60 mg/k.g BW *n*-docosanol. The organs were collected at 1, 3 and 5 days post the first *n*-docosanol dosage injection. The titer of recovered NDV was determined by qRT-PCR. The letters a, b and c indicate a significant difference in the viral log10 value between the low, medium, and high *n*-docosanol dosage groups at the same time point (*value of p* of <0.05). The asterisk indicates a significant difference in the viral log10 value between the untreated group and the high or medium or low dosage group at the same time point (*p* < 0.01). ns represents the non-significant value.

The effect of *n*-docosanol on virus load in the lymphatic (spleen), digestive (proventriculus), respiratory (lungs), and nervous (brain) tissues was measured for each group at days 1, 3, and 5 post the first *n*-docosanol administration by qRT-PCR. In all the treated groups, the highest virus titer (from 3.9 ± 0.2 to 6.4 ± 0.6 log_10_/g tissue) was detected in the spleen which was relatively close to that appeared in the digestive tissue (from 3.5 ± 0.3 to 6.4 ± 0.3 log_10_/g tissue). In the respiratory tissue, virus titer was found to be less (from 1.6 ± 0.4 to 5.9 ± 0.4 log_10_/g tissue), however, the lowest titer (from 0 to 4.47 ± 0.13 log_10_/gm tissue) was detected in the nervous tissue (Figure 4). In the untreated group, virus load in the spleen, digestive and respiratory tissues reached its maximum level at day 3 post the first *n*-docosanol treatment, and then started to decline. In contrast, in the treated groups, the virus titer was found to reduce significantly and gradually in the successive days post the first dosage treatment until it reached its lowest level when the treatment course was completely finished (Figures 4A–C). Nevertheless, virus load increased gradually over time in the brain tissue following the first dosage treatment. Contrary to the medium- and high-dosage treated groups that showed no significant increase in virus load in the brain tissue over the time post the first dosage injection, a significant increase in virus load was detected in both the untreated- and the low-dosage treated groups (Figure 4D). Overall, the three treated groups showed a significant reduction in virus load in all the tested tissues as compared to the untreated group. Although, the low-dosage treated group showed significantly less reduction in virus load than both high and medium treated groups which showed non-significant variation in the reduction of virus load between each other (Figure 4).

### NDV-related pathological damages reduced post *n*-docosanol treatment

**Figure 5:**
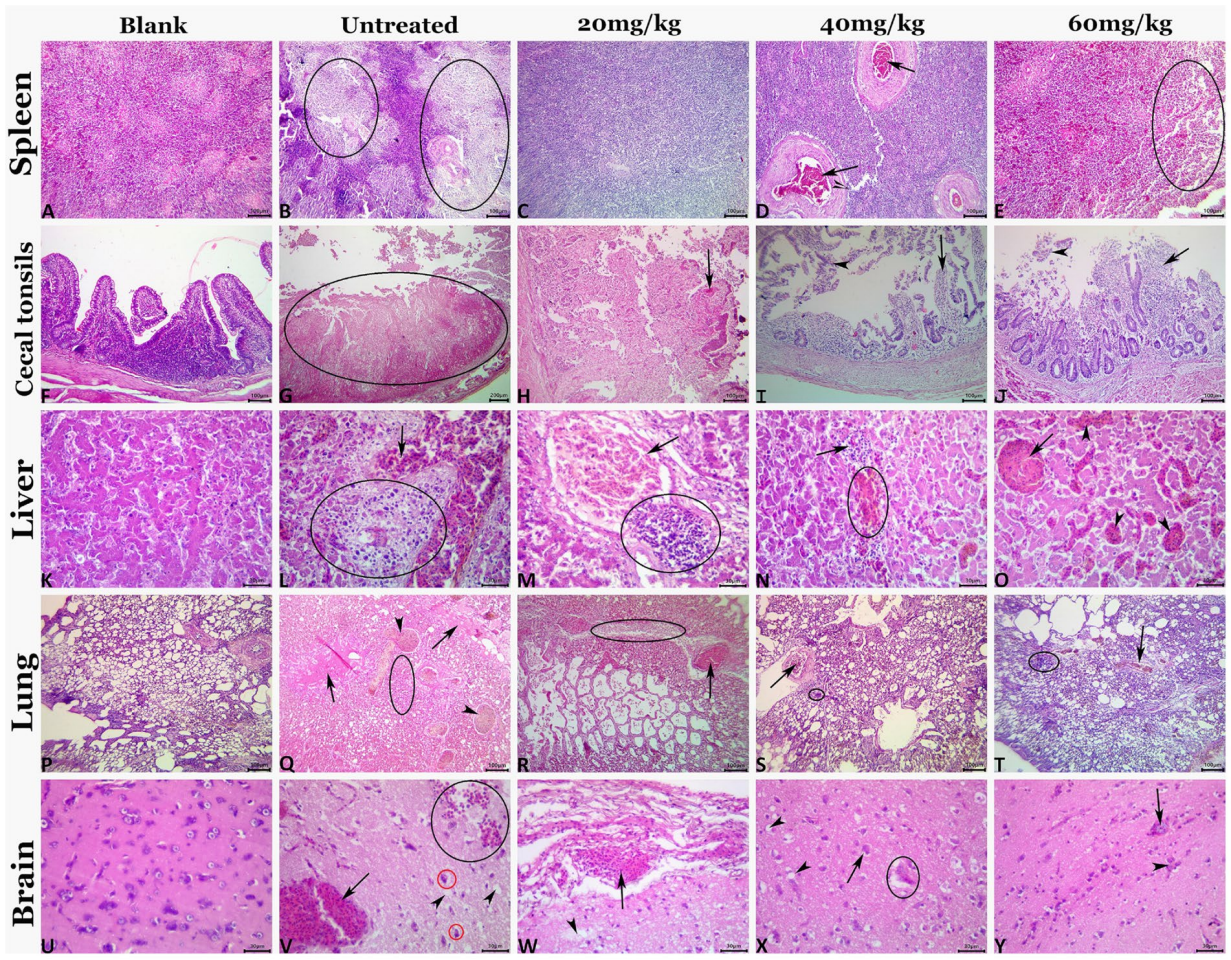
Representative photomicrograph of H&E-stained tissue sections demonstrating histopathological alterations in experimentally NDV infected chicken groups treated with 20, 40, and 60 mg/kg n-docosanol. The blank panel shows normal histology for all tissues of the control negative chickens. The NDV-infected untreated panel shows lymphoid depletion (ellipses) in the splenic tissue (B), mucosal coagulative necrosis (ellipse), and luminal hemorrhagic content in cecal tonsils (G), notable vascular congestion (arrow), and focal area of coagulative necrosis infiltrated with inflammatory cell infiltrate (ellipse) in the hepatic tissue (L), prominent vascular congestion (arrowhead), pulmonary edema (arrows) and inflammatory cell infiltrates (ellipse) in the pulmonary tissue (Q), and notable vascular congestion (arrow), neuronal pyknosis with perineural vacuolation (red ellipses), hemorrhage (black ellipse), and neuropil microcavitation (arrowheads) in the brain tissue (V). The 20 mg/kg panel shows diffuse lymphoid depletion in the spleen (C), mucosal coagulative necrosis with hemorrhage (arrow) in cecal tonsils (H), portal congestion (arrow), and focal mononuclear cell infiltrate (ellipse) in hepatic tissue (M), congestion (arrow), and focal mononuclear cell infiltrate (ellipse) in pulmonary tissue (R), meningeal congestion (arrow), and neuropil microcavitation (arrowhead) in the brain (W). The 40 mg/kg panel shows vascular congestion (arrows) in the spleen (D), mucosal mononuclear cell infiltration (arrow) with epithelial desquamation (arrowhead) in cecal tonsils (I), minute hemorrhage (ellipse) and mononuclear cell infiltrations (arrow) in hepatic tissue (N), congestion (arrow), and minute mononuclear cell infiltration (ellipse) in pulmonary tissue (S), and neuronal pyknosis with perineural vacuolation (arrow), neuropil microcavitation (arrowhead), and neuronophagia (ellipse) (X). The 60 mg/kg panel shows focal lymphoid depletion (ellipse) in the spleen (E), mucosal mononuclear cell infiltration (black arrow) with epithelial desquamation (arrowhead) in cecal tonsils (J), almost normal histological picture except for sinusoidal dilatations (arrowheads), and vascular congestion (arrow) in hepatic tissue (O), congestion (arrow), and focal mononuclear cell infiltrate (ellipse) in pulmonary tissue (T), and congestion (arrow), and glial clustering (arrowhead) in the brain (Y).

The highly virulent NDV infections almost cause severe lesions in various organs. Therefore, histopathological scoring was conducted to evaluate the pathological changes and to check whether *n*-docosanol treatment could mitigate the lesions within the tissues of different NDV targeted organs. All the examined tissues of the vehicle control birds showed normal histological findings (Figure 5; Blank). As expected, the untreated birds manifested massive histological alterations including the different cells within all the examined tissues after NDV infection, (Figure 5; Untreated). We found that treatment with *n*-docosanol at all dosages (20, 40, and 60 mg/kg) significantly diminished both the frequencies and severities of the ND-induced tissues’ lesions. These histopathological findings were reduced to some extent after the treatment with the low dosage of *n*-docosanol (Figure 5; 20 mg/kg), while much less histological alterations were detected in the high- and medium-dosage treated groups (Figure 5; 40 and 60 mg/kg). A quantitative lesion scoring for the histological alterations of all examined tissues among all groups was summarized in Table 2.

**Table 2:** Effect of 20, 40, and 60 mg/k.g BW *n*-docosanol on the frequency and severity of NDV-induced histopathological lesions in different body tissues of experimentally infected chickens.

**Organ**	**Lesion**	**Untreated**		**20 mg/kg**		**40 mg/kg**		**60 mg/kg**	
		FQ	SV	FQ	SV	FQ	SV	FQ	SV
**Spleen**	Necrosis	64	1.4 ± 1.1	34	1.0 ± 1.3	20	0.7 ± 0.6	22	0.8 ± 0.4
	Depletion	76	3.4 ± 1.5	52	2.1 ± 0.8	48	1.2 ± 0.7	45	0.9 ± 0.3
	Congestion	88	4.1 ± 0.8	72	1.9 ± 0.8	68	1.1 ± 0.7	64	1.0 ± 0.8
	Hemorrhage	24	1.3 ± 0.8	12	0.8 ± 0.6	12	0.1 ± 0.3	12	0.1 ± 0.3
	Hemosiderosis	24	1.2 ± 0.7	12	0.6 ± 0.5	12	0.1 ± 0.3	12	0.1 ± 0.3
**Cecal tonsils**	Necrosis	72	2.8 ± 1.5	46	1.1 ± 1.1	22	0.6 ± 0.8	18	0.3 ± 0.5
	Hemorrhage	70	1.9 ± 1.0	42	1.2 ± 1.0	18	0.4 ± 0.7	20	0.4 ± 0.5
	Epithelial desquamation	100	4.4 ± 0.7	92	2.9 ± 0.8	70	1.7 ± 0.8	67	1.3 ± 0.4
	Congestion	96	2.3 ± 0.8	76	1.2 ± 0.6	52	0.5 ± 0.7	52	0.6 ± 0.7
	Leukocytic infiltration	86	1.6 ± 1.1	52	0.9 ± 0.8	20	0.8 ± 0.7	18	0.7 ± 0.8
**Liver**	Leukocytic infiltration	60	4.1 ± 0.7	36	2.5 ± 0.5	16	1.5 ± 0.5	12	1.1 ± 0.3
	Congestion	94	4.3 ± 0.5	74	3.6 ± 0.5	52	2.5 ± 0.5	48	1.9 ± 0.5
	Hemorrhages	38	1.2 ± 0.4	14	0.6 ± 0.5	10	0.1 ± 0.3	8	0.1 ± 0.3
	Cellular swelling	58	4.6 ± 0.5	42	3.2 ± 0.7	22	2.3 ± 0.5	24	2.4 ± 0.5
	Vacuolations	46	4.4 ± 0.5	30	2.8 ± 0.6	12	1.6 ± 0.5	10	0.8 ± 0.4
	Nuclear pyknosis	42	4.1 ± 0.7	28	2.0 ± 0.6	18	0.9 ± 0.5	18	1.3 ± 0.8
	Single cell necrosis	38	2.2 ± 1.3	26	1.2 ± 0.6	12	0.4 ± 0.5	16	0.1 ± 0.3
	Focal coagulative necrosis	20	1.5 ± 0.9	14	0.3 ± 0.5	6	0.1 ± 0.3	8	0.2 ± 0.4
**Lung**	Leukocytic infiltration	48	4.3 ± 0.5	31	3.1 ± 0.7	22	1.9 ± 0.7	16	1.3 ± 0.6
	Congestion	49	3.3 ± 0.5	37	2.4 ± 0.5	21	1.6 ± 0.7	18	1.5 ± 0.5
	Hemorrhages	21	1.2 ± 0.7	11	0.3 ± 0.5	5	0.1 ± 0.3	3	0.1 ± 0.3
	Septal thickening	41	2.7 ± 0.9	23	1.2 ± 0.4	13	0.4 ± 0.5	9	0.5 ± 0.7
	Alveolar edema	33	2.2 ± 0.4	19	0.8 ± 0.7	10	0.3 ± 0.5	7	0.2 ± 0.4
	Alveolar emphysema	20	0.9 ± 0.8	16	0.5 ± 0.8	8	0.4 ± 0.7	4	0.1 ± 0.3
	Pulmonary consolidation	16	0.5 ± 0.7	9	0.2 ± 0.4	5	0.1 ± 0.3	2	0.1 ± 0.3
	Coagulative necrosis	12	0.8 ± 0.7	8	0.2 ± 0.4	6	0.2 ± 0.4	2	0.2 ± 0.4
**Brain**	Cerebral congestion	24	1.8 ± 0.7	18	1.5 ± 0.5	12	0.3 ± 0.5	10	0.3 ± 0.5
	Meningeal congestion	22	1.0 ± 0.8	12	0.7 ± 0.5	8	0.2 ± 0.4	8	0.3 ± 0.5
	Hemorrhages	12	0.9 ± 0.8	8	0.3 ± 0.5	4	0.2 ± 0.4	4	0.1 ± 0.3
	Neuronal shrinkage	66	1.8 ± 0.7	42	0.8 ± 0.6	22	0.4 ± 0.5	16	0.3 ± 0.5
	Perineural vacuolation	60	1.9 ± 0.9	40	0.6 ± 0.8	18	0.5 ± 0.5	12	0.3 ± 0.5
	Neuronal necrosis	14	0.6 ± 0.7	8	0.3 ± 0.5	6	0.2 ± 0.4	4	0.1 ± 0.3
	Neuronophagia	8	0.3 ± 0.5	4	0.2 ± 0.4	4	0.1 ± 0.3	2	0 ± 0
	Gliosis	38	2.2 ± 0.7	22	1.2 ± 0.4	16	0.6 ± 0.5	12	0.4 ± 0.5
	Perivascular cuffing	16	0.7 ± 0.6	10	0.2 ± 0.4	8	0.2 ± 0.4	2	0.1 ± 0.3

FQ is frequency in percent and SV is severity. Values are represented as the mean ± SE. The means within the same row carrying different superscripts are significant at *p* < 0.05.

### *n*-docosanol treatment significantly reduced virus shedding in cloacal and oropharyngeal secretions

**Figure 6:**
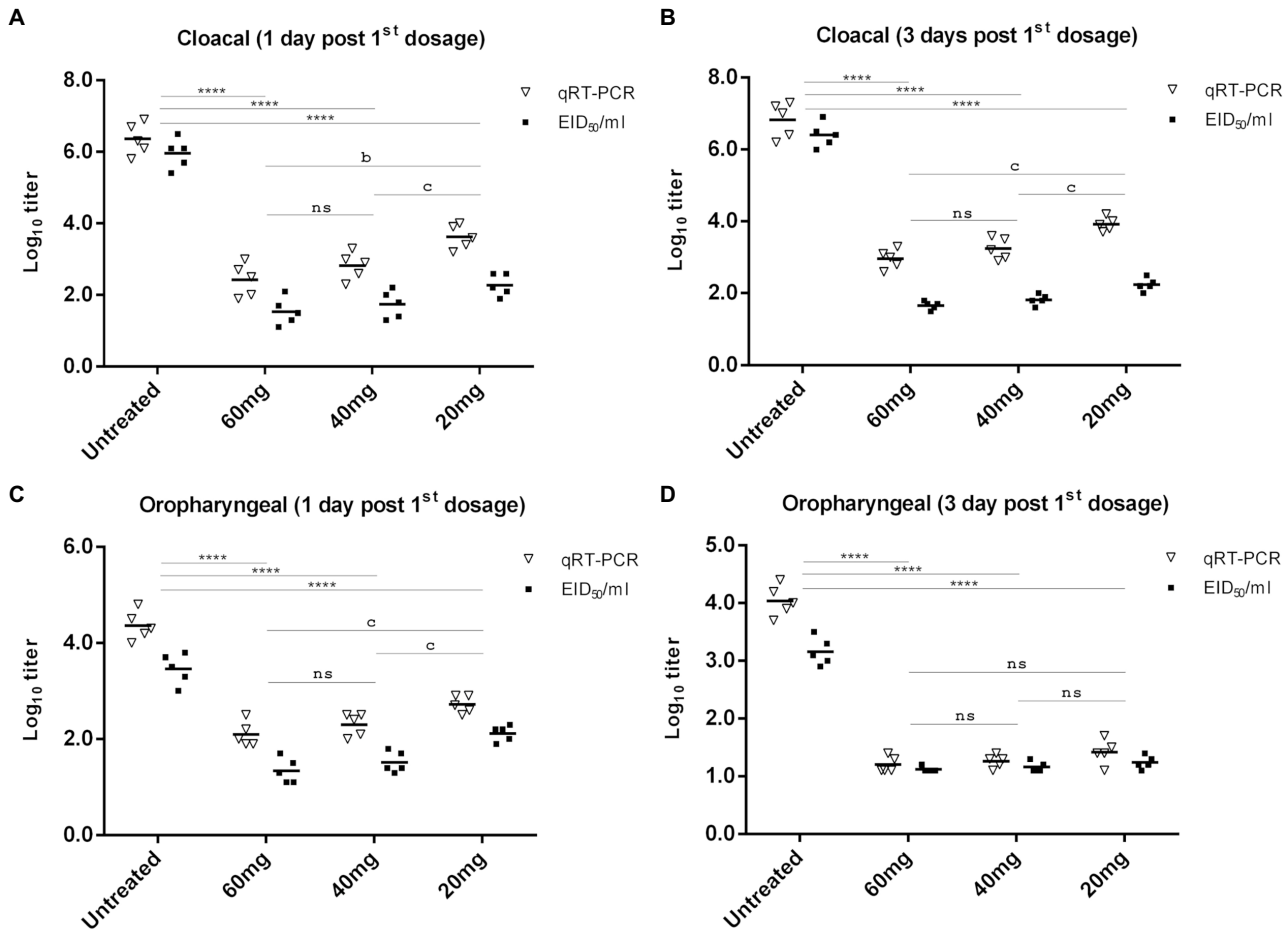
NDV cloacal **(A,B)** and oropharyngeal **(C,D)** shedding in experimentally infected (10^6^ EID_50_) chickens one and 3 days after treatment with 20, 40, and 60 mg/k.g BW *n*-docosanol. The titer of recovered NDV was determined by qRT-PCR and the infectivity assay using ECE. The asterisk indicates a significant difference in the viral log10 value between the untreated group and the high or medium or low dosage group at the same time point (*p* < 0.01) and error bars included. ns represents the non-significant value.

Virus shedding was evaluated by titrating the number of viruses recovered from both the oropharyngeal and cloacal swabs on days 1, 3, 5, and 7 post-first *n*-docosanol administration using qRT-PCR and the infectivity (EID_50_) assay. In all the treated groups, higher NDV titer was recovered from the cloacal swabs than that from the oropharyngeal ones at all the time points (Figure 6). The qRT-PCR-based NDV titer was found to be higher than the EID_50_-based titer in all the groups at all time points (Figure 6).

In the untreated group, virus titers of approximately 10^6.36^ and 10^6.82^ EID_50_/ml (by qRT-PCR), and 10^5.96^ and 10^6.4^ EID_50_/ml (by the infectivity assay in ECE) were determined in the cloacal swabs on days 1 and 3 post-infection, respectively (Figures 6A,B). Lower virus titer of 10^4.36^ and 10^4.04^ EID_50_/ml (by qRT-PCR), respectively, and 10^3.46^ and 10^3.16^ EID_50_/ml (by the infectivity assay in ECE) was detected in the oropharyngeal swabs from days 1 and 3 post infection, respectively (Figures 6C,D). A significant reduction in virus titer recovered from both cloacal and oropharyngeal swabs was detected in the groups treated with *n*-docosanol, as compared with the untreated group (Figure 6). Virus titer from the cloacal swabs on day 1 post first treatment was found to be relatively close to that on day 3 post-first treatment. The group treated with the low dosage of *n*-docosanol showed significantly higher virus titer than the medium and high dosage groups where a non-significant difference in virus titer was detected between the two groups (Figures 6A,B). From the oropharyngeal swabs collected on day 1 post the first treatment, chickens treated with the low dosage of *n*-docosanol showed significantly higher virus titers than in the chickens treated with medium and high dosages (Figure 6C). However, on day 3 post the first treatment, low virus titer with non-significant variation between the groups was determined in the treated chickens (Figure 6D). On day 5 post the first treatment, a low qRT-PCR mean virus titer (10^1.4^ EID_50_/ml) was detected only in the low dosage-treated group, while no virus was detected in the oropharyngeal swabs in all the treated groups, neither by qRT-PCR nor by the infectivity assay. There were no sufficient survivors in the untreated group on day 5 to evaluate the virus shedding.

## Discussion

For decades ND has stroke the global poultry industry causing drastic economic losses, especially in developing and tropical countries (Yusoff and Tan, 2001). Since there is no antiviral drug for ND, the only available control measure is vaccination. However, due to the high mutation nature of NDV, new variants are continuously emerging and cause several outbreaks annually, even in vaccinated chickens (Miller et al., 2009). Therefore, the development of anti-ND remedies would be a complementary or alternative approach for the efficient control of the disease. *n*-Docosanol is a saturated aliphatic alcohol with antiviral properties which has been approved by the Food and Drug Administration (FDA) as a medication against human viral cold sores (Abreva, 2018). Its synthesis endogenously in the human body with its high safety margin (Johnson, 1988; Harvey, 1989; Katz et al., 1991), makes *n*-docosanol surpass many other antiviral therapeutics that are used for veterinary purposes.

There is evidence that *n*-docosanol is effective in the treatment of already-established viral infections (Katz et al., 1991). In the present study, we tested the therapeutic effect of *n*-docosanol on NDV infection and virus shedding in broiler chicks artificially challenged with a highly virulent NDV. To simulate the authentic situation, we intended to evaluate how effective *n*-docosanol was against ND when administered shortly after emerging the disease’s symptoms in the infected chickens. Since the NDV infection is characterized by its rapid progression and sick birds, especially severelyinfected ones, cannot reach the food and water supplies, *n*-docosanol was administered by intramuscular injection to ensure both quick effect and precise dosage uptake. We started monitoring the therapeutic effects of *n*-docosanol for 12 days, from the first-day post the first dosage treatment to track the progress of *n*-docosanol curative effect after each dosage and after the completion of the treatment course.

Consistent with the normal incubation period of the velogenic ND ranging mostly between 2 and 7 days (Parede and Young, 1990), the disease’s symptoms began to appear 51-h post-infection, and then raised in a rapid progressive manner in untreated control group until all the chickens died on day 9 post-infection (Figures 1, 2), as expected (Ferreira et al., 2019). Unlike other reported compounds with no or low therapeutic effects on virus infection *in vivo* (Mtambo et al., 1999; Hassanin et al., 2020), *n*-docosanol was found to largely increase both curative and survival rates by around 50% in the treated chickens, especially in the medium and high dosages. Interestingly, no significant variation in the curative and survival rates was observed between the treatments with the medium and high dosages of *n*-docosanol. This can be due to a kind of cell saturation with *n*-docosanol at a concentration between the medium and large dosages. In addition, the high degree of *n*-docosanol association with living cells (Katz et al., 1994) would potentiate and prolong its action on NDV-infected cells. A significant improvement in both curative and survival rates was observed 24 h post the first dosage administration and was enhanced by the subsequent dosages (Figures 2, 3) indicating the immediate effect of *n*-docosanol. An explanation is that *n*-docosanol reached its peak uptake per cell in a short time. On the cultured Vero cells, it was noticed that within 6–12 h post-exposure, *n*-docosanol reaches its peak uptake (Katz et al., 1994). Highly concentrated docosanol inside living human dermal fibroblasts and human epidermal keratinocytes has been also detected 24 h after drug treatment (You et al., 2017).

The velogenic NDV is transported *via* the blood to the body organs for late replication, after the initial replication in the entry site (Maclachlan and Dubovi, 2016). Therefore, virus load in NDV-targeted body tissues is believed to be a predictor of viral growth and a metric for evaluating the inhibitory impact of *n*-docosanol. The high and medium dosages of *n*-docosanol showed a close significant reduction in NDV titers in the tested tissues (Figure 4). Twenty-four hours post the first dosage administration, *n*-docosanol inhibitory effect was found to be higher in the spleen and proventriculus than in the lung (Figures 4A–C). However, NDV titer in both tissues appeared higher than that of the lung tissue throughout the treatment course. This could be attributed to the viscerotropic nature of the virus. In addition, the infection of the spleen and proventriculus by the velogenic NDV mostly started later than in the lung (Hussein et al., 2019), which in turn changed the virus growth curve in tested tissues. More investigation is required to test whether cells in the spleen and proventriculus are more or less responsive to *n*-docosanol than the cells in the lung and other organs. In the case of brain tissue, virus load and consequently the neurological signs were found to progress slower. The neurotropic velogenic NDV is almost slower in onset and the affected chickens developed the neurological lesions at 5 days post-infection (dpi) or later (Brown et al., 1999; Ecco et al., 2011). Since the applied virus was viscerotropic, the neurological signs and lesions as well as the virus load in the brain were the lowest among the tested tissues and reached their peak later (7 dpi). However, the effect of the high and medium dosages of *n*-docosanol was remarkable one day after the first treatment, with no NDV detected in the brain tissue (Figure 4D).

For evaluating the therapeutic effect of *n*-docosanol at the cellular level, extensive histopathological scoring (Hussein et al., 2018) was performed on the NDV-targeted organs. Consistent with other studies (Cattoli et al., 2011; Zenglei and Liu, 2015; Mahmoud et al., 2019), we also observed severe lesions in the spleen and cecal tonsils post-infection with velogenic viscerotropic VIId NDV. We found substantial minimal organs’ lesions in chickens treated with *n*-docosanol, indicating that *n*-docosanol could slow down histopathological alterations induced by NDV infection. High *n*-docosanol dosage showed the highest rescue effect in the virus-infected tissues, however, the effect was found to be not significantly higher than the medium dosage (Table 2; Figure 5).

NDV is excreting in a high concentration in the body secretions and feces of infected chickens. Besides, virus stability and its wide host range facilitate viral lateral transmission between different flocks (Maclachlan and Dubovi, 2016). Therefore, the reduction of NDV shedding is considered an ultimate target for the control of ND. The high and medium dosages of *n*-docosanol showed a significant decrease in the NDV shedding in both rectal and oropharyngeal swabs 1 and 3 days post the first dosage administration (Figure 6). Surprisingly, the NDV was detected neither in cloacal nor in oropharyngeal swabs 5 and 7 days post the first dosage in the chickens treated with high and medium *n*-docosanol dosages. From epidemiological insight, using *n*-docosanol as a remedy in NDV-infected flocks will shorten the state of the viral carriage during the convalescence period and consequently minimize the liability of viral spread to healthy flocks. In general, there was a lower NDV titer in all the cloacal and oropharyngeal swabs when the virus was titrated by the infectivity assay in ECE than by qRT-PCR. The higher sensitivity of qRT-PCR could be a reason. However, we believe that *n*-docosanol reduced the titer of infectious NDVs less than the recorded qRT-PCR virus titer and this extra virus titer in qRT-PCR was the number of genomes of non-infectious NDVs. The tight association of *n*-docosanol to the infected cells (Katz et al., 1994) or critical interactions with lipids in the viral envelope as suggested by Katz et al. (1991) could be the reason for the existence of these incomplete non-infectious NDVs. Therefore, we recommend the use of infectivity assays than qRT-PCR for precise evaluation of NDV shedding after treatment by *n*-docosanol or even other antivirals.

## Conclusion

In summary, the data revealed substantial therapeutic activity of *n*-docosanol against the velogenic NDV *in vivo*, indicating its potential as a candidate drug for the treatment of NDV infection in chickens. Moreover, the ability of *n*-docosanol to considerably reduce NDV shedding enables its application in the control strategy of ND. Furthermore, the non-significant variation between the effect of both dosages 40 and 60 mg/kg BW of *n*-docosanol on the velogenic NDV infection and shedding open the gate for future studies on the precise *n*-docosanol dosage of the threshold therapeutic effect on ND. A further investigation is also recommended for the evaluation of the therapeutic effect of *n*-docosanol in vaccinated chickens and also to find out the mechanism/s, including the immunomodulatory effects by which *n*-docosanol can inhibit NDV.

## Data availability statement

The original contributions presented in the study are included in the article/supplementary material, further inquiries can be directed to the corresponding authors.

## Ethics statement

The animal study was reviewed and approved by the Zagazig University − Institutional Animal Care and Use Committee (ZU-IACUC) under the approval number ZU-IACUC/2/F/128/2020.

## Author contributions

AO designed, supervised, and completed the experiments and wrote the manuscript. AH assisted in experimental design and work. AS and AMM assisted in experiments. HM helped in manuscript write up and data analysis. MM completed the histopathology experiment and its data analysis. ASM assisted in the experimental supervision and manuscript revision. All authors contributed to the article and approved the submitted version.

## Funding

AO is funded by a post-doctorate fellowship (CDM-2020) from the Ministry of Higher Education of the Arab Republic of Egypt.

## Conflict of interest

The authors declare that the research was conducted in the absence of any commercial or financial relationships that could be construed as a potential conflict of interest.

## Publisher’s note

All claims expressed in this article are solely those of the authors and do not necessarily represent those of their affiliated organizations, or those of the publisher, the editors and the reviewers. Any product that may be evaluated in this article, or claim that may be made by its manufacturer, is not guaranteed or endorsed by the publisher.
